# The Draft Genome Sequence of *Nocardioides* sp. Strain CF8 Reveals the Scope of Its Metabolic Capabilities

**DOI:** 10.1128/genomeA.00439-13

**Published:** 2013-07-05

**Authors:** Jeffrey A. Kimbrel, Jeff Chang, Daniel J. Arp, Luis A. Sayavedra-Soto

**Affiliations:** Department of Botany and Plant Pathology, Oregon State University, Corvallis, Oregon, USAa; Molecular and Cellular Biology Program, Oregon State University, Corvallis, Oregon, USAc; Center for Genome Research and Biocomputing, Oregon State University, Corvallis, Oregon, USAd; Joint BioEnergy Institute, Lawrence Berkeley National Laboratory, Emeryville, California, USAb

## Abstract

*Nocardioides* sp. strain CF8 was isolated from a soil sample collected at the Hanford Department of Energy site, Richland, WA. The strain was identified in microcosms based on its ability to grow on butane and has been characterized for its potential applications in the biodegradation of halogenated hydrocarbons. Here, the draft genome sequence is reported.

## GENOME ANNOUNCEMENT

*Nocardioides* sp. strain CF8 is a high-G+C aerobic Gram-positive bacterium belonging to the lineage *Actinobacteria*. This bacterium was enriched from hydrocarbon-contaminated core material from the Hanford Department of Energy (DOE) site in Washington state and subsequently isolated using butane (C_4_H_10_) as the sole carbon source for growth (1). CF8 has potential applications in schemes for the bioremediation of sites contaminated with halogenated hydrocarbons (2, 3). CF8 can grow under heterotrophic conditions, as well as those with C_2_ to C_16_ *n*-alkanes as the sole carbon source (1). Moreover, *Nocardioides* sp. strain CF8 is the first Gram-positive bacterium reported to use a particulate butane monooxygenase (pBMO) to grow on C_2_ to C_6_ *n*-alkanes. The pBMO monooxygenase represents a deeply branched third lineage in the groups of ammonia monooxygenases (AMO) (from the ammonia-oxidizing bacteria), and the particulate methane monooxygenases (pMMO) (from the methane-oxidizing bacteria) (4).

The genome of *Nocardioides* sp. strain CF8 was sequenced using paired-end sequencing on an Illumina genome analyzer IIx (Illumina, Inc., CA) at the Center for Genome Research and Biocomputing at Oregon State University. The sequence reads (average length of 80 bp) were *de novo* assembled using Velvet version 0.7.55 (5) into 165 contigs that were >200 bp in length. A high-quality draft genome sequence was derived by reordering and coalescing the contigs into 21 scaffolds using the mauveAligner program (6) and the *Nocardioides* SJ614 genome (accession no. NC_008699.1) as a reference. The size of the CF8 genome was estimated to be 4,204,777 bases in length, with approximately 32× coverage. There are a total of 4,003 coding genes (coding sequence features without a pseudoqualifier) that represent 3,759,514 bases (G+C content, 69.9%) for a density of 0.952 genes per kilobase (1,050 bases per gene). Several transposase- and mutator-type coding sequences could not be ordered correctly and were simply reported at the end of the genome sequence. Annotation was based on the xBASE bacterial genome annotation service (http://www.xbase.ac.uk/annotation/) and a secondary automatic genome annotation performed by the Center for Genome Research and Biocomputing at Oregon State University (ETA xbase pipeline). The manual analysis of the genome composition was performed using Artemis Sanger Institute software (7).

In addition to previously characterized genes and enzyme activities for hydrocarbon utilization (e.g., pBMO and a binuclear-iron-containing AlkB*-*type alkane monooxygenase), the genome contains genes that potentially encode a second AlkB-type monooxygenase and four aromatic-ring (hydroxylating and cleavaging) dioxygenases. Preliminary analyses of the draft genome sequence of *Nocardioides* sp. strain CF8 reveal its potential for applications in bioremediation. The sequence is useful for genome-enabled approaches to study the full scope of substrates that this organism is capable of using (6).

### Nucleotide sequence accession numbers.

The genome sequence was deposited at DDBJ/EMBL/GenBank under the accession no. ASEP00000000.1. The version described in this paper is the first version, accession no. ASEP01000000 (BioProject PRJNA60007).
